# Deep Learning-Based Intelligent Sorting of Potato Tubers and Mineral Impurities: System Development and Experimental Evaluation

**DOI:** 10.3390/foods15122070

**Published:** 2026-06-08

**Authors:** Qian Wang, Ke Chen, Qiying Li, Qiuying Xu, Weigang Deng

**Affiliations:** College of Mechanical and Electrical Engineering, Inner Mongolia Agricultural University, Hohhot 010018, China

**Keywords:** potato tuber, mineral impurity sorting, soil clod, stone, YOLOv10n-PB, real-time sorting

## Abstract

To improve the efficiency, accuracy, and operational stability of postharvest potato tuber sorting in the presence of mineral impurities, mainly soil clods and stones, an intelligent sorting system for potato tubers and mineral impurities was designed and developed. The system employed YOLOv10n as the baseline network and incorporated a PSA module together with a dynamic blur augmentation strategy to establish a task-adapted detection model, termed YOLOv10n-PB. Rather than treating detection accuracy alone as the optimization objective, the proposed system jointly considered detection performance, inference-latency stability, temporal–spatial coordination, and pneumatic rejection reliability. In addition, a programmable logic controller and pneumatic actuators were integrated to enable online target identification and dynamic removal. Comparative experiments involving lightweight YOLO models and $L_{25}(5^{3})$ orthogonal tests were conducted to evaluate the effects of conveyor belt speed, material spacing, and classification threshold on sorting performance. The results showed that YOLOv10n-PB achieved a mAP@0.5 of 98.9% on the test set. Among the investigated factors, conveyor belt speed had the greatest effect on overall sorting accuracy, followed by material spacing and classification threshold. Range analysis identified the optimal parameter combination as a conveyor belt speed of 0.2 m/s, a material spacing of 9 cm, and a classification threshold of 0.4. Validation experiments under these conditions yielded an overall sorting accuracy of 98.3%, a combined mineral-impurity removal accuracy of 98.3%, and a potato tuber false rejection rate of 1.7%. These results demonstrate the feasibility of the proposed system for accurate and stable automatic sorting of potato tubers and mineral impurities under postharvest operating conditions.

## 1. Introduction

Potato is one of the most important food crops worldwide. With the widespread adoption of mechanized harvesting, harvesting efficiency has been substantially improved. However, under complex field conditions, mineral impurities, mainly soil clods and stones, are often collected together with potato tubers during mechanized harvesting [1]. These mineral impurities differ from organic residues, biological defects, and chemical contaminants because they are hard, irregularly shaped, and mechanically abrasive. If they are not removed in a timely manner, they may cause impact damage, surface abrasion, blockage, and equipment wear during conveying, cleaning, grading, storage, and subsequent processing. Soil-clod removal and potato damage control are therefore closely related in postharvest handling and harvesting-device design [2]. Therefore, the accurate and real-time removal of mineral impurities from potato tubers is important for improving postharvest handling efficiency, reducing tuber damage, and advancing intelligent potato processing.

In recent years, machine vision, optical sensing, depth sensing, hyperspectral imaging, and deep learning have been increasingly applied to potato identification, grading, defect detection, and impurity removal. RGB-D-based dynamic identification has shown that three-dimensional visual information can improve potato recognition and cleaning under moving conditions [3]. Commercial optical sorting systems have also been applied to postharvest potato handling. For example, the TOMRA 3A optical sorter is designed for unwashed potatoes and uses optical sensing to remove foreign materials, including stones and soil clods, from harvested root crops [4]. Recent potato grading studies have further integrated machine vision with mechanical devices to improve grading accuracy and operational efficiency [5]. Hyperspectral imaging combined with machine learning has also been used for non-destructive detection of external potato defects, indicating the potential of spectral information for distinguishing visually similar targets [6]. In addition, R-CNN-based potato segmentation has been applied in sorting processes, demonstrating that deep-learning-based visual recognition can support automated potato handling [7]. These studies indicate that visual sensing and deep learning provide useful technical support for postharvest potato sorting and mineral-impurity removal.

In addition to visual recognition, recent studies have emphasized the importance of real-time sorting mechanisms, actuator execution, and system-level integration. Automated sorting mechanisms have shown that actuator design, conveyor speed, sorting accuracy, and repeatability are important factors affecting practical sorting performance [8]. Robotic seed potato sorting systems have further demonstrated the potential of integrating visual detection, localization, tracking, and execution mechanisms for potato sorting applications [9]. However, these studies have mainly focused on recognition performance, mechanical separation, grading accuracy, segmentation accuracy, or device-level sorting effectiveness. The coordinated influence of inference-latency stability, temporal–spatial rejection-error estimation, PLC-based timing control, and pneumatic rejection on dynamic sorting accuracy remains insufficiently discussed.

Another important challenge in dynamic sorting is motion-induced image degradation. In online object detection, motion blur can reduce target boundary clarity and affect recognition stability, especially when objects move rapidly through the imaging region [10]. Therefore, dynamic potato tuber–mineral impurity sorting requires not only accurate visual recognition, but also lightweight and temporally stable object detection. YOLO-based detectors have been widely used because of their balance between detection accuracy and deployment efficiency [11]. Recent reviews of the Ultralytics YOLO series have further shown that YOLO models have evolved toward faster, lighter, and more deployment-oriented object detection frameworks [12]. Newer YOLO variants have also improved real-time detection performance through more efficient network structures and training strategies [13]. However, in agricultural sorting systems, model selection is often still evaluated mainly by detection accuracy or average inference speed, whereas latency fluctuation and its effect on downstream actuator execution are rarely quantified.

Despite these advances, several limitations remain. First, existing studies have focused mainly on static or quasi-dynamic performance indicators, such as recognition accuracy, segmentation performance, grading accuracy, or device-level sorting effectiveness, whereas the influence of inference-latency fluctuation on dynamic sorting accuracy has received limited attention. In high-speed sorting systems, fluctuations in algorithm inference time may lead to spatiotemporal desynchronization among target recognition, control decision-making, and actuator execution, thereby reducing rejection accuracy. Second, many existing models have been trained and validated under relatively controlled experimental conditions, and their robustness under practical disturbances, such as motion blur, soil adhesion, target overlap, and rapid target movement, has not been sufficiently verified. Third, although some studies have reported integrated sorting devices, the coordinated optimization of recognition performance, temporal stability, control response, and actuator execution remains inadequate. As a result, current potato tuber–mineral impurity sorting systems still suffer from limited robustness, insufficient real-time dynamic performance, and restricted generalization ability under complex operating conditions.

To address these issues, the purpose of this study was to develop and experimentally evaluate a real-time dynamic sorting system specifically for separating potato tubers from mineral impurities, mainly soil clods and stones, rather than for detecting biological defects, organic residues, or chemical contaminants. The specific objectives were to: (1) establish an engineering model describing the relationship between inference-latency fluctuation and rejection-position deviation; (2) select and improve a lightweight detection model by jointly considering detection accuracy, inference speed, and temporal stability; (3) integrate machine vision, PLC-based control, and pneumatic rejection into a functional sorting prototype; and (4) optimize the operating parameters affecting dynamic sorting performance using orthogonal experiments. Compared with previous agricultural machine-vision and real-time sorting studies, the present study emphasizes system-level coordination under dynamic conveyor conditions. The methodological contribution of this work does not rely solely on modifying the YOLO architecture. Instead, it lies in the task-oriented integration of YOLOv10n-PB with inference-latency stability evaluation, temporal–spatial rejection-error estimation, PLC-based timing control, pneumatic rejection, and orthogonal optimization of operating parameters. This integrated framework links visual recognition performance with downstream actuator execution, which is essential for real-time mineral-impurity removal from potato tubers.

## 2. Materials and Methods

### 2.1. Collection of Potato Tuber and Mineral Impurity Samples and Dataset Construction

Three sample categories were collected to construct the dataset: potato tubers, soil clods, and stones. In this study, soil clods and stones were defined as mineral impurities because they are the main hard foreign materials introduced during mechanized potato harvesting and postharvest conveying. Potato tubers were selected from a locally cultivated yellow-fleshed commercial potato cultivar grown in Inner Mongolia, China. The material represented commercial potato tubers commonly used in local postharvest handling and sorting operations. Because the experiment focused on the visual separation of potato tubers from mineral impurities rather than on cultivar-dependent quality evaluation, intact tubers with average commercial size and typical yellow-fleshed appearance were used as target samples.

Soil clods and stones were collected from potato-growing fields in the same production area as representative mineral impurities commonly mixed with potato tubers during mechanized harvesting and postharvest conveying. The soil clods used in this study were hard, compacted, and irregularly shaped field aggregates, with surface color and texture similar to soil-adhered potato tubers. These characteristics made them representative and visually challenging mineral impurities for evaluating the proposed sorting system. Samples were selected so that the maximum projected length of each potato tuber, soil clod, and stone ranged from 4 to 8 cm, as measured manually before image acquisition. To cover different surface states, both clean and soil-adhered samples were included. Clean potato tubers were washed and kept at room temperature for 24 h before image acquisition, whereas unwashed potato tubers, soil clods, and stones with adhered soil were collected to simulate complex operating conditions.

Images were acquired using a Hikrobot MV-CS200-10GC industrial camera (Hikrobot Technology Co., Ltd., Hangzhou, China) mounted vertically above the conveyor belt at a distance of approximately 50 cm. A constant-intensity LED light source was used to reduce the influence of ambient light variation. The image resolution was 5472 × 3648 pixels. The dataset contained five representative image groups: clean potato tubers (700 images), soil-adhered or fully covered potato tubers (612 images), stones (622 images), soil clods (721 images), and mixed scenes containing potato tubers and mineral impurities (800 images), yielding a total of 3455 original images. These five image groups accounted for 20.3%, 17.7%, 18.0%, 20.9%, and 23.2% of the original dataset, respectively, indicating that no single image group dominated the dataset. The dataset covered several major visual variations relevant to potato tuber–mineral impurity sorting, including clean tuber surfaces, soil-adhered or fully covered tubers, irregular clod and stone morphology, and mixed scenes containing multiple target types. However, image acquisition was conducted under controlled laboratory conditions using a fixed top-view camera, constant LED illumination, and an enclosed dark-box structure; therefore, uncontrolled field illumination and industrial disturbances were not fully represented. Because soil clods and stones had irregular shapes and high sensitivity to illumination, and because their color and texture could resemble those of soil-adhered potato tubers, target discrimination was challenging. In addition, conveyor operation at 0.5 m/s introduced noticeable motion blur, further increasing the difficulty of visual recognition.

**Figure 1:**
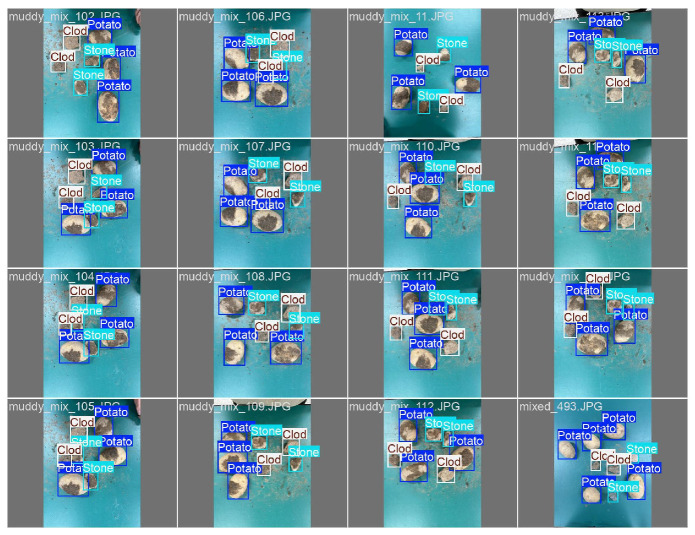
Example of ground-truth bounding-box annotation for potato tubers and mineral impurities using LabelImg. Source: Authors’ own work.

All images were manually annotated using LabelImg, and the ground-truth bounding boxes were defined by the minimum enclosing rectangles of the targets in YOLO format $\left(class,x_{center},y_{center},width,height\right)$ (Figure 1). The dataset was divided into training, validation, and test sets at a ratio of 8:1:1. To improve model robustness under complex operating conditions, data augmentation strategies, including color jitter, random occlusion, Mosaic augmentation, and dynamic blur simulation, were applied only to the training set. In particular, dynamic blur simulation was used to mimic image trailing during conveyor operation by applying Gaussian kernels of 5×5 and 7×7. After augmentation, the training set was expanded to 4310 images, whereas the validation and test sets retained their original distributions [10].

### 2.2. Temporal–Spatial Coupling Model

In a dynamic sorting system for potato tubers and mineral impurities, the detection model is required, not only to achieve high recognition accuracy but also to maintain adequate temporal synchronization with the physical rejection mechanism. Because the samples move continuously on the conveyor belt, the time delay between visual detection and actuator execution directly affects the spatial accuracy of target rejection.

Let v denote the conveyor speed and L denote the horizontal distance from the camera detection position to the pneumatic rejection point. Under ideal conditions, the flight time required for a target to travel from the detection position to the rejection position can be expressed as

(1) $$t_{\mathrm{flight}}=\frac{L}{v}$$

where L is the horizontal distance between the center of the detection region and the pneumatic rejection position, and v is the conveyor speed. In the present system, the camera was mounted approximately 500 mm above the conveyor belt, and the horizontal distance from the center of the detection region to the pneumatic rejection station was 450 mm. Therefore, L was set to 0.45 m.

In practice, the visual detection and rejection process includes multiple delay components, including model inference delay ($t_{\mathrm{infer}}$), communication delay between the host computer and the programmable logic controller (PLC) ($t_{\mathrm{comm}}$), PLC processing delay ($t_{\mathrm{PLC}}$), and pneumatic actuator response delay ($t_{\mathrm{cylinder}}$). Accordingly, the trigger advance time required by the control system can be written as

(2) $$t_{\mathrm{advance}}=t_{\mathrm{infer}}+t_{\mathrm{comm}}+t_{\mathrm{PLC}}+t_{\mathrm{cylinder}}$$

and the actual trigger time should satisfy

(3) $$t_{\mathrm{trigger}}=t_{\mathrm{flight}}-t_{\mathrm{advance}}$$

To avoid treating the delay of each module as a fixed constant, the major delay terms were measured experimentally under identical operating conditions, and the results are summarized in Table 1.

From the perspective of system control, the trigger advance time $t_{\mathrm{advance}}$ should be determined on the basis of the combined mean delay of all modules. However, for model comparison, $t_{\mathrm{comm}}$, $t_{\mathrm{PLC}}$, and $t_{\mathrm{cylinder}}$ are mainly determined by the communication hardware, control program, and actuator characteristics, and therefore remain nearly constant across different detection models. Consequently, the effect of $t_{\mathrm{infer}}$ and its fluctuation on dynamic rejection accuracy was emphasized in the subsequent analysis.

In industrial control systems, the mean inference delay $t_{\mathrm{infer}}$ is commonly used as the time-compensation parameter. When the inference delay of an individual frame deviates from this mean value, a temporal error is introduced:

(4) $$\Delta t=t_{\mathrm{infer}}-{\bar{t}}_{\mathrm{infer}}$$

Assuming constant conveyor motion, this temporal error can be further converted into a spatial deviation of the rejection position, ΔX, as follows:

(5) $$\Delta X=v\cdot \Delta t=v\cdot (t_{\mathrm{infer}}-{\bar{t}}_{\mathrm{infer}})$$

Equation (5) indicates that the spatial rejection error is linearly related to both the conveyor speed and the fluctuation in inference delay. Therefore, even when the average inference delay satisfies the real-time requirement, insufficient temporal stability may still lead to a spatial mismatch between detection and actuation, thereby reducing rejection precision and operational stability at higher conveyor speeds.

It should be noted that Equation (5) is an engineering approximation established under several simplifying assumptions, including constant conveyor speed within a short time window, a fixed distance between the detection and rejection positions, and the dominance of longitudinal displacement in determining rejection accuracy. Under these assumptions, fluctuations in inference delay can be approximately transformed into rejection-position deviations along the conveyor direction. This model was used to analyze the error mechanism in dynamic sorting and to support model selection, rather than to represent the full execution error of the actual system.

Taking conventional YOLOv8n as an example, at a conveyor speed of 0.5 m/s, if the inference-delay fluctuation is ±1.5 ms (standard deviation: 0.5 ms, corresponding to ±3σ), the corresponding spatial deviation is

(6) ΔX=0.5×(±1.5 ms)=±0.75 mm

For small targets with a width of approximately 30–40 mm, such as small stones, a positional deviation of ±0.75 mm already accounts for a non-negligible proportion of the target width and may cause the airflow action point to deviate from the target center, thereby reducing rejection stability. By contrast, the inference-delay fluctuation of YOLOv10n was ±0.3 ms (standard deviation: 0.1 ms), and the corresponding spatial deviation was

(7) ΔX=0.5×(±0.3 ms)=±0.15 mm

The theoretical spatial deviation was therefore markedly reduced, which is beneficial for improving the spatial matching between actuator action and target position. These results indicate that, in dynamic sorting applications, model selection should consider not only detection accuracy and average inference speed but also inference-delay stability.

Considering the action range of the pneumatic rejection mechanism and the spatial tolerance required for small targets, this study used a small spatial error range as a reference criterion for evaluating delay stability. At a conveyor speed of 0.5 m/s, according to Equation (5), maintaining the spatial deviation within ±0.5 mm requires the inference-delay fluctuation to be controlled within ±1.0 ms. It should be emphasized that this threshold is an engineering reference derived under the conditions of the present system and was used mainly for model selection and relative comparison rather than as a universal threshold. It should also be noted that the predicted positional errors derived from Equation (5) were not directly measured under all conveyor-speed conditions in this study. Therefore, the temporal–spatial coupling model should be interpreted as a first-order engineering estimation for explaining the influence of inference-latency fluctuation on rejection-position deviation.

### 2.3. Model Selection and Training Strategy

#### 2.3.1. Model Selection Criteria and Evaluation Protocol

To evaluate the suitability of different object detection models for dynamic sorting, several mainstream lightweight YOLO models were tested under identical hardware and inference settings. The tests were performed on an RTX 3060 platform using TensorRT FP16 inference with an input resolution of 640×640, a batch size of 1, and 1000 consecutive inference frames. For each model, the mean inference latency and its standard deviation were calculated. The theoretical positional error reported in Table 1 was derived from inference-latency fluctuation according to Equation (5) and was used to characterize the theoretical spatial deviation that each model may introduce in dynamic control scenarios.

As shown in Table 2, the candidate models differed markedly in both mean inference latency and latency stability. For dynamic sorting systems, fluctuations in inference latency can be translated into deviations in rejection position; therefore, mean inference speed alone is insufficient for evaluating real-time deployment performance. Under the test conditions used in this study, YOLOv10n achieved a mean inference latency of 2.2 ms and a latency standard deviation of 0.1 ms, corresponding to a theoretical positional error of ±0.15 mm, which satisfied the spatial positioning requirement of the present system. By contrast, all other candidate models exceeded the engineering reference range defined in this study.

#### 2.3.2. Task-Adapted YOLOv10n-PB Model and Unified Training Settings

YOLOv10n comprises a Backbone, Neck, and Head for feature extraction, multi-scale fusion, and target prediction, respectively. Unlike conventional detectors that rely on non-maximum suppression (NMS), YOLOv10n adopts an end-to-end prediction mechanism, thereby reducing temporal uncertainty during inference and making it suitable for dynamic sorting tasks. To adapt YOLOv10n to the visual characteristics of dynamic potato tuber–mineral impurity sorting, a partial self-attention (PSA) module and a dynamic blur augmentation strategy were introduced as task-oriented model-level and data-level modifications, respectively. Specifically, the PSA module was inserted after the final high-level feature extraction stage of the YOLOv10n backbone and before the neck feature-fusion module. This placement was used to enhance global contextual feature representation while preserving the lightweight structure and real-time inference capability of the original YOLOv10n model. The PSA module was used to enhance the representation of local discriminative features, which is important for distinguishing soil-adhered potato tubers, soil clods, and stones with similar color and texture. Dynamic blur augmentation was used to improve robustness to image degradation caused by conveyor motion. Accordingly, three YOLOv10n-based variants were constructed for ablation analysis: the baseline model (YOLOv10n-B), the PSA-enhanced variant (YOLOv10n-P), and the PSA plus dynamic-blur-augmentation variant (YOLOv10n-PB). It should be noted that YOLOv10n-PB was designed as a task-adapted lightweight detection model for the proposed dynamic sorting system, rather than as a fundamentally new object-detection architecture.

To ensure comparability in the subsequent ablation study, all YOLOv10n-based variants were trained under identical settings. The default composite loss was adopted, including bounding-box regression loss, classification loss, and distribution focal loss, with CIoU used for box regression [7]. The initial learning rate was set to 0.01, stochastic gradient descent with momentum was used as the optimizer, the momentum coefficient was 0.937, the weight decay was 0.0005, the batch size was 32, and the number of training epochs was 300. During training, box loss, classification loss, distribution focal loss, and validation mAP@0.5 were monitored, and early stopping was triggered at approximately epoch 215.

### 2.4. Sorting System Configuration and Control Architecture

#### 2.4.1. Overall Configuration and Workflow

**Figure 2:**
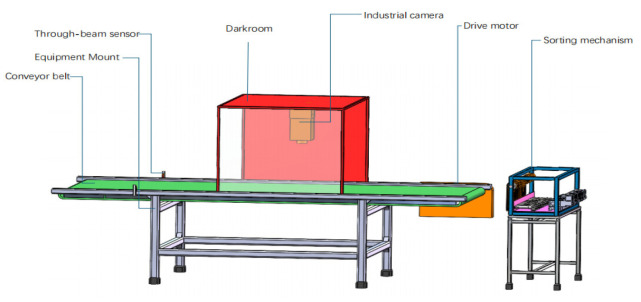
Intelligent sorting equipment for potato tubers and mineral impurities. Source: Authors’ own work.

The intelligent sorting of potato tubers and mineral impurities system mainly consisted of a conveying unit, a frame, an industrial vision acquisition module, a photoelectric trigger sensor, a drive motor, a pneumatic rejection unit, and a control system, as shown in Figure 2. In this configuration, the conveyor belt served as the material transport unit, the vision module was used for target acquisition, the photoelectric sensor acted as the trigger unit, the upper computer and programmable logic controller (PLC) constituted the control core, and the pneumatic actuator functioned as the terminal rejection unit. These modules were integrated on a unified working platform to enable online identification and dynamic sorting of potato tubers, soil clods, and stones. The overall dimensions of the prototype were 2475 mm × 790 mm × 1060 mm, with a total motor power of 650 W and belt-driven transmission.

**Figure 3:**
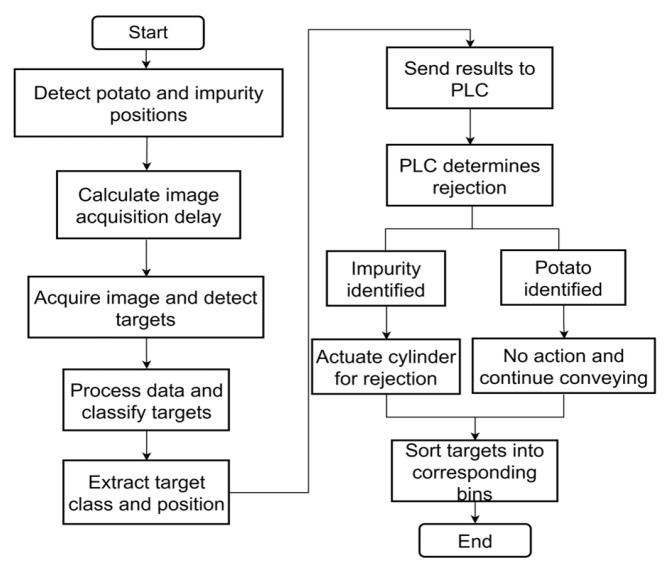
Workflow of the dynamic sorting system for potato tubers and mineral impurities. Source: Authors’ own work.

During operation, potato tubers and mineral impurities were transported into the detection region by the conveyor belt. A front-mounted photoelectric sensor generated a trigger signal when a target entered the acquisition zone, and the industrial camera captured a single-frame image according to the preset delay calculated from the conveyor speed and the relative position between the sensor and the imaging region. The acquired image was transmitted to the upper computer, where the YOLOv10n-PB model identified the target category and extracted its position information. The recognition result was then sent to the PLC, which performed timing calculation according to the conveyor speed and the distance between the detection point and the rejection station. When the target reached the corresponding rejection position, the pneumatic actuator was triggered to remove the impurity target. The overall workflow can be summarized as target triggering, image acquisition, target recognition, information transmission, timing coordination, and pneumatic rejection, as illustrated in Figure 3.

#### 2.4.2. Vision Acquisition and Control Modules

**Figure 4:**
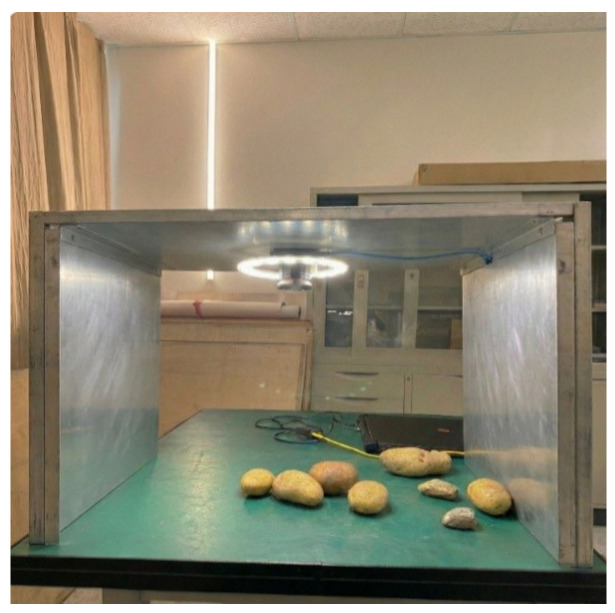
Industrial vision acquisition module. Source: Authors’ own work.

The vision acquisition module consisted of an industrial camera, an LED area light source, an enclosed dark-box structure, and an image acquisition platform, as shown in Figure 4. An MV-CS200-10GC industrial camera (Hangzhou Hikrobot Digital Technology Co., Ltd., Hangzhou, China) was used for top-view imaging. To minimize the effect of ambient light variation, the acquisition region was illuminated by an LED area light source and enclosed within a dark-box structure, thereby providing a relatively uniform and stable illumination environment for image acquisition. This configuration improved the visibility of target edges and surface texture features and provided reliable image input for model training and online deployment.

**Figure 5:**
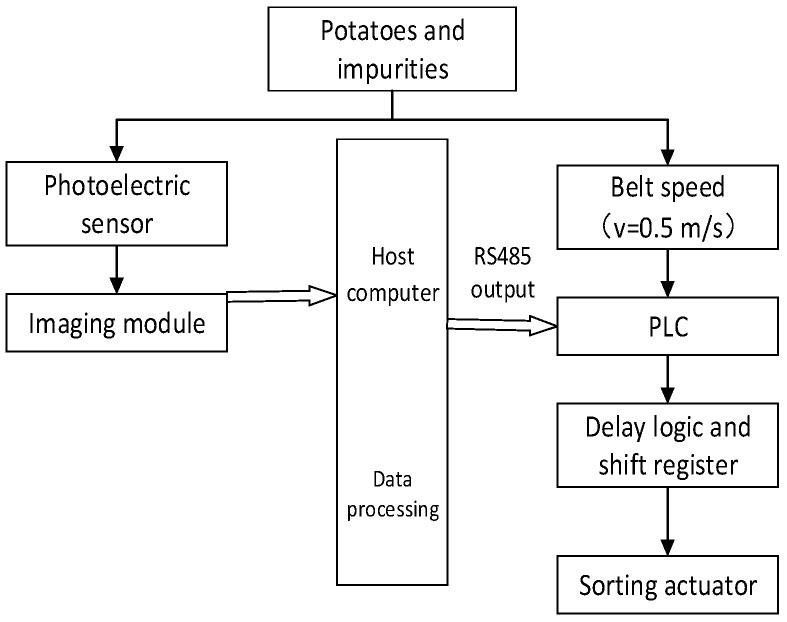
Hardware architecture of the control system. Source: Authors’ own work.

The control module consisted of an upper computer, a PLC, a front-mounted photoelectric sensor, the industrial camera, a drive motor, and an air compressor, as shown in Figure 5. When the photoelectric sensor detected a target entering the imaging region, it triggered the camera to capture an image. The acquired image was processed by the upper computer using YOLOv10n-PB, and the recognition result was transmitted to the PLC through an RS485 interface. The PLC then performed timing coordination according to the conveyor speed and the distance between the detection and rejection positions, and triggered the pneumatic actuator to remove impurity targets at the corresponding location.

#### 2.4.3. Conveying and Pneumatic Rejection Mechanism

**Figure 6:**
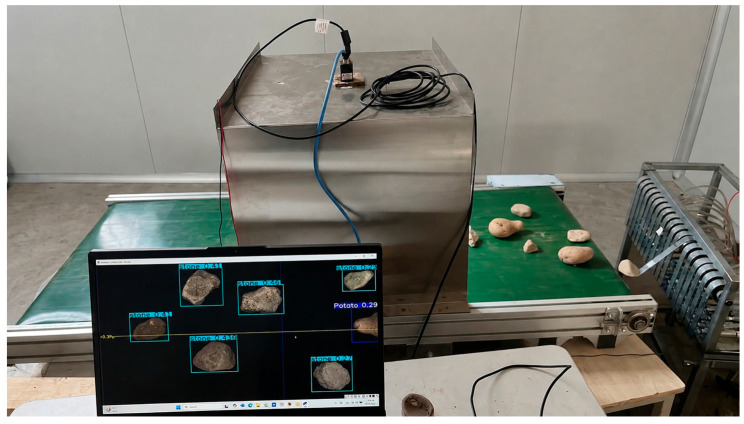
Conveying and sorting mechanism for potato tubers and mineral impurities. Source: Authors’ own work.

The conveying and rejection mechanism mainly consisted of a conveyor belt, a stepper motor, a pneumatic cylinder array, finger flippers, and an air compressor, as shown in Figure 6. This subsystem enabled continuous transport of potato tubers and mineral impurities and location-specific rejection according to the control commands generated by the vision and PLC modules. After target recognition, the PLC performed timing coordination according to the conveyor speed and the relative distance between the detection and rejection positions, and then triggered the corresponding actuator to remove impurity targets.

The conveyor system employed a GlobalBelt GB-1500 belt with an effective width of 600 mm and a length of 1500 mm. The belt was driven by an 86BYG250H stepper motor with a rated power of 650 W, and the adjustable speed range was 0–0.6 m/s.

The pneumatic rejection system used 20 AIRTAC MA16 double-acting cylinders arranged in parallel across the conveyor width. Each cylinder had a stroke of 25 mm, a bore diameter of 16 mm, a nozzle diameter of 6 mm, and a center-to-center spacing of 30 mm, with the array installed 240 mm above the belt surface. The system operated at 0.5 MPa and provided full-width coverage of the 600 mm conveyor. To enhance rejection performance, stainless-steel finger flippers (150 mm × 20 mm × 2 mm) were installed in front of the nozzles to assist in removing irregular targets such as soil clods and stones.

### 2.5. Orthogonal Experimental Design and Validation Protocol

To optimize the operating parameters of the proposed sorting system under representative dynamic sorting conditions, an orthogonal experimental design was adopted. Based on the adjustable range of the equipment, material feeding conditions, and model deployment settings, conveyor speed, material spacing, and classification threshold were selected as the experimental factors. Conveyor speed mainly affected the temporal coordination among target detection, information transmission, and pneumatic actuation; material spacing mainly affected target occlusion, boundary adhesion, and adjacent-target interference; and the classification threshold mainly influenced the trade-off between false rejection and missed detection. Considering the equipment operating range and representative dynamic working conditions, the levels of conveyor speed were set to 0.2, 0.3, 0.4, 0.5, and 0.6 m/s, the levels of material spacing were set to 1, 3, 5, 7, and 9 cm, and the levels of classification threshold were set to 0.3, 0.4, 0.5, 0.6, and 0.7.

An $L_{25}(5^{3})$ orthogonal array was used to design the combined parameter experiments. Each parameter combination in the L25 orthogonal array was tested once, and the experiment was used primarily for engineering parameter screening. The optimal parameter combination identified from the orthogonal analysis was further evaluated in an independent validation experiment using 120 samples, including 60 potato tubers, 35 soil clods, and 25 stones. The comprehensive impurity-rejection accuracy $\left(Y_{1}\right)$ and the overall sorting accuracy $\left(Y_{2}\right)$ were used as the primary evaluation indices, together with the potato false rejection rate for overall performance assessment. The two main indices were defined as

(8) $$Y_{1}=\frac{N_{TP}}{N_{TP}+N_{FP}}\times 100\%$$

(9) $$Y_{2}=\frac{N_{TP}+N_{TN}}{N_{TP}+N_{FP}+N_{TN}+N_{FN}}\times 100\%$$

Here, $N_{TP}$ denotes the number of correctly rejected mineral impurities, $N_{FP}$ denotes the number of falsely rejected potatoes, $N_{TN}$ denotes the number of correctly retained potatoes, and $N_{FN}$ denotes the number of missed mineral impurities. The factor levels used in the orthogonal design are listed in Table 3.

To further evaluate the relative effects of the operating parameters, analysis of variance (ANOVA) was conducted on the orthogonal experimental results. Conveyor speed, material spacing, and classification threshold were treated as independent factors, whereas comprehensive mineral-impurity rejection accuracy and overall sorting accuracy were used as response variables. For each factor, the sum of squares, mean square, F value, *p* value, and contribution ratio were calculated. A significance level of *p* < 0.05 was used. Because the orthogonal experiment was primarily designed for engineering parameter screening and each parameter combination was tested once, the ANOVA results were interpreted as an exploratory assessment of factor contributions rather than as a confirmatory statistical test based on repeated trials.

To verify the effectiveness of the optimal parameter combination identified from the orthogonal analysis, an independent validation experiment was conducted under laboratory conditions using the same equipment and sample conditions. In the validation test, conveyor speed, material spacing, and classification threshold were set according to the selected optimal combination, and potato false rejection rate, comprehensive impurity-rejection accuracy, and overall sorting accuracy were recorded as validation indices.

## 3. Results

### 3.1. Latency Stability and Comparative Performance of Candidate YOLO Models

Table 2 compares the latency stability and deployment-related theoretical positional error of candidate lightweight YOLO models under identical hardware and inference settings. The results show that the candidate models differed markedly in mean inference latency, latency fluctuation, and the corresponding theoretical positional error. YOLOv10n achieved the lowest mean inference latency (2.2 ms) and the smallest latency standard deviation (0.1 ms), corresponding to a theoretical positional error of only ±0.15 mm. This value satisfied the spatial positioning requirement of the present system, whereas all other candidate models exceeded the engineering reference range.

**Figure 7:**
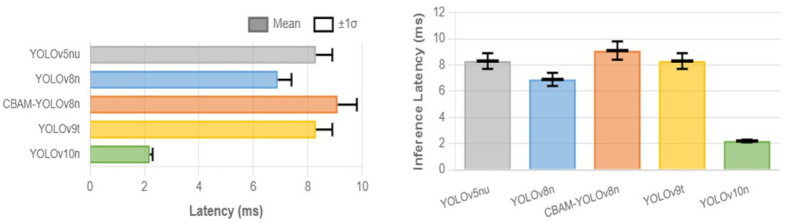
Comparison of Average Inference Latency and Stability Across Different YOLO Models. Source: Authors’ own work.

Figure 7 further illustrates the differences in mean inference latency and temporal stability among the candidate models. The error bars represent the dispersion of inference latency and provide a direct measure of model stability during continuous operation. Consistent with the results in Table 2, YOLOv10n showed the smallest latency fluctuation among the compared models.

To further illustrate the differences in inference latency and temporal stability among models, the mean inference latency and fluctuation range of each model are compared in Figure 7. The error bars represent the dispersion of inference latency and were used to characterize the temporal stability of each model under continuous operation.

After the latency-stability analysis, the detection performance of the candidate models was further compared under identical dataset partitioning and training settings. As shown in Table 4, the models exhibited different trade-offs between detection performance and deployment efficiency. YOLOv9t achieved the highest mAP@0.5, whereas YOLOv10n provided the fastest inference speed while maintaining a mAP@0.5 of 98.9%. With a relatively small parameter size, YOLOv10n showed favorable deployment efficiency. When considered together with the latency-stability results, these findings indicate that YOLOv10n offered the most suitable overall balance between detection accuracy, inference efficiency, and dynamic-control adaptability.

Therefore, YOLOv10n was selected as the backbone network for subsequent model enhancement, and the PSA module together with the dynamic blur augmentation strategy was introduced to construct the final deployment model, YOLOv10n-PB.

### 3.2. Ablation Study and Training Convergence of YOLOv10n-Based Variants

Table 5 presents the ablation results of different YOLOv10n-based variants. The baseline model, YOLOv10n-B, achieved a mAP@0.5 of 97.6% with an inference speed of 470 f·s^−1^. After introducing the PSA module, mAP@0.5 increased to 98.3%, whereas inference speed decreased slightly to 455 f·s^−1^. When dynamic blur augmentation was further incorporated, the resulting YOLOv10n-PB variant achieved a mAP@0.5 of 98.9% with an inference speed of 450 f·s^−1^. These results indicate that the PSA module and dynamic blur augmentation improved detection performance under complex dynamic conditions while preserving real-time inference capability. Therefore, YOLOv10n-PB was selected as the final deployment model for the proposed real-time sorting system. The improvement should be interpreted as a task-oriented enhancement for dynamic potato tuber–mineral impurity sorting, rather than as a standalone architectural breakthrough.

**Figure 8:**
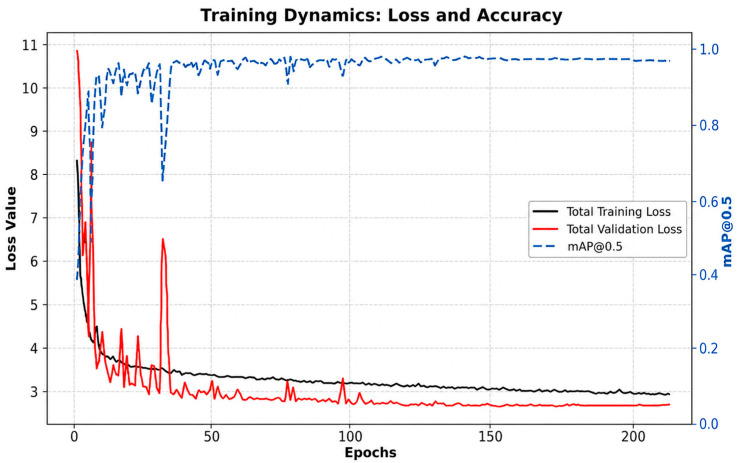
Changes in training loss and detection accuracy for the YOLOv10n-PB model. Source: Authors’ own work.

Figure 8 shows the training convergence behavior of the selected YOLOv10n-PB model. During training, box loss, classification loss, distribution focal loss, and validation mAP@0.5 were monitored. The losses decreased steadily, whereas validation mAP@0.5 increased progressively before reaching a stable plateau. Early stopping was triggered at approximately epoch 215, indicating stable convergence under the present dataset and training settings.

### 3.3. Class-Level Detection Performance of YOLOv10n-PB

**Figure 9:**
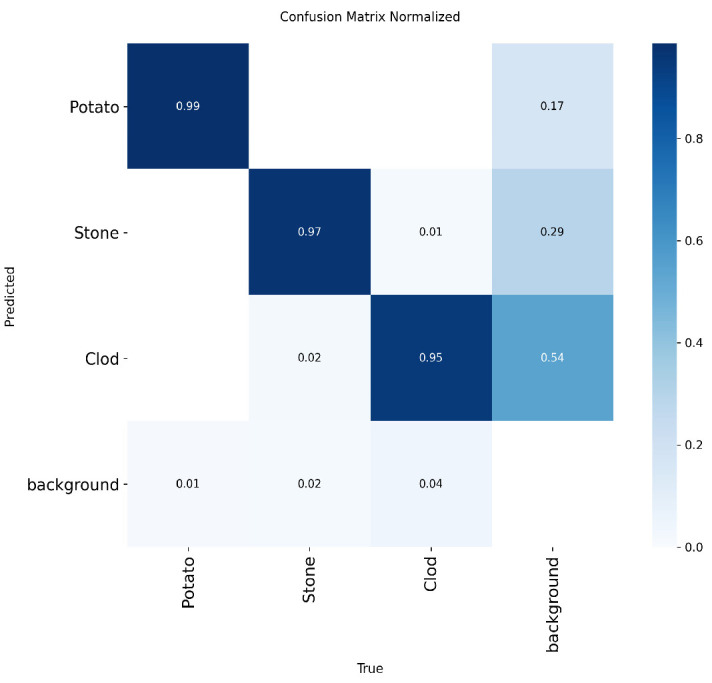
Normalized confusion matrix of YOLOv10n-PB on the test set. Source: Authors’ own work.

To further evaluate class-level recognition performance, the normalized confusion matrix of YOLOv10n-PB on the test set is shown in Figure 9. The model achieved high recognition accuracy for all three target categories, namely potato tubers, soil clods, and stones. The latter two categories represented the mineral impurities investigated in this study. Recognition of potato samples was relatively stable, whereas limited confusion remained between soil clods and stones. This misclassification was likely associated with irregular morphology, variable surface texture, and soil adhesion in some samples. Nevertheless, the overall classification results indicate that YOLOv10n-PB is suitable for online recognition of potato tubers and mineral impurities in the proposed sorting system.

### 3.4. Orthogonal Experimental Results and Parameter Optimization

#### 3.4.1. Orthogonal Test Outcomes

The orthogonal test results are summarized in Table 6. The results show that the sorting performance varied considerably across different parameter combinations. In general, lower conveyor speed and larger material spacing were associated with higher overall sorting accuracy and higher comprehensive impurity-rejection accuracy, whereas the classification threshold mainly influenced the trade-off between potato false rejection and impurity missed detection. As conveyor speed increased, the overall sorting accuracy decreased. By contrast, increasing material spacing generally improved both overall sorting accuracy and comprehensive impurity-rejection accuracy. A higher classification threshold tended to reduce false rejection of potatoes, but at the cost of increased missed detection of some impurity targets.

#### 3.4.2. Range Analysis and Optimal Parameter Combination

The range analysis results are presented in Table 7. Based on the mean values of overall sorting accuracy, the influence of the three factors followed the order of conveyor speed (A) > material spacing (B) > classification threshold (C). According to the range analysis, the statistically optimal combination was identified as A1B5C2, corresponding to a conveyor speed of 0.2 m/s, a material spacing of 9 cm, and a classification threshold of 0.4. However, this combination was not directly included in the $L_{25}(5^{3})$ orthogonal array. Therefore, an additional validation experiment was conducted to verify the effectiveness of this parameter combination under the same equipment and sample conditions.

#### 3.4.3. ANOVA and Factor Contribution Analysis

To further strengthen the statistical interpretation of the orthogonal experimental results, ANOVA was performed for the two response variables: overall sorting accuracy and comprehensive mineral-impurity rejection accuracy. The results are shown in Table 8. For overall sorting accuracy, conveyor speed, material spacing, and classification threshold all showed significant effects under the additive orthogonal model (*p* < 0.001). The contribution ratios followed the order of conveyor speed (38.94%) > material spacing (34.21%) > classification threshold (24.53%), which was consistent with the range analysis.

For comprehensive mineral-impurity rejection accuracy, the classification threshold showed the largest contribution ratio (59.41%), followed by conveyor speed (19.83%) and material spacing (19.61%). This result indicates that conveyor speed mainly affected the overall dynamic sorting stability, whereas the classification threshold had a stronger influence on the rejection of mineral impurities. Therefore, the operating parameter selection should consider both overall sorting accuracy and mineral-impurity rejection accuracy rather than relying on a single response index.

### 3.5. Validation of the Optimal Parameter Combination

To verify the effectiveness of the optimal parameter combination identified from the range analysis, an independent validation experiment was conducted under laboratory conditions using the same equipment and sample conditions. According to the range-analysis result, the operating parameters were set to a conveyor speed of 0.2 m/s, a material spacing of 9 cm, and a classification threshold of 0.4. The validation results are summarized in Table 9.

As shown in Table 9, under the optimal parameter combination, the potato tuber false rejection rate was 1.7%, the rejection accuracy for soil clods was 97.1%, and the rejection accuracy for stones reached 100.0%. The corresponding comprehensive impurity-rejection accuracy and overall sorting accuracy were both 98.3%, confirming the effectiveness of the parameter combination identified by the range analysis.

## 4. Discussion

### 4.1. Significance of Latency Stability in Dynamic Sorting

The present study focused on mineral impurities, mainly soil clods and stones, rather than on biological defects, organic residues, or chemical contaminants. This distinction is important because mineral impurities are hard, irregularly shaped, and mechanically abrasive. During postharvest conveying and processing, they may cause tuber bruising, surface abrasion, equipment wear, blockage, and instability in subsequent grading operations.

The present study indicates that latency stability is a critical but often overlooked factor in the dynamic sorting of potato tubers and mineral impurities. Previous studies have mainly emphasized detection accuracy and average inference speed, whereas in dynamic rejection systems, fluctuations in inference latency can be translated into spatial deviations between target recognition and actuator execution [17]. Under the present test conditions, YOLOv10n showed the lowest mean inference latency and the smallest latency fluctuation among the candidate models, corresponding to a theoretical positional error of ±0.15 mm, which satisfied the engineering reference range of the proposed system. These results suggest that model selection for dynamic sorting should consider not only detection accuracy and deployment efficiency but also temporal stability, particularly when recognition outputs are tightly coupled with downstream control and actuator timing. Compared with recent studies on agricultural machine vision and real-time sorting systems, the present work places greater emphasis on the coupling between visual recognition and actuator execution. Xu and Lu [8] designed and evaluated pneumatically powered online sweetpotato sorting mechanisms and demonstrated the importance of actuator design, conveyor speed, sorting accuracy, and repeatability in real-time sorting systems. Lyu et al. [9] developed a robotic seed potato sorting system based on a monocular two-stage ROI-guided detection framework, showing the potential of integrating visual detection, tracking, localization, and robotic execution for potato sorting. However, these studies mainly focused on sorting mechanism performance, visual detection, localization, or robotic execution. In contrast, the present study links lightweight detection, inference-latency stability, temporal–spatial rejection-error estimation, PLC-based timing control, and pneumatic rejection within a single prototype system. Therefore, the contribution of this work lies not only in improving detection accuracy but also in evaluating how recognition stability affects real-time mineral-impurity removal from potato tubers.

The validation experiment achieved an overall sorting accuracy of 98.3% under the optimal laboratory-scale operating condition. This result indicates that the proposed prototype has the potential to provide accurate mineral-impurity removal under controlled dynamic conveying conditions. However, direct numerical comparison with commercial sorting systems should be interpreted cautiously because commercial systems are designed for industrial-scale throughput, multi-product adaptability, long-term stability, and broader quality-control tasks. For example, the TOMRA 5B is a commercial belt sorter used for potato products and other food materials, with emphasis on sensor-based inspection, quality control, and yield improvement. Similarly, the SiftAI system from KPM Analytics is designed for AI-based potato sorting, sizing, grading, and defect or foreign-material detection in commercial processing lines. Compared with these mature industrial platforms, the present system is a laboratory-scale prototype focused specifically on the real-time separation of potato tubers from mineral impurities, mainly soil clods and stones. Therefore, the 98.3% accuracy should be regarded as evidence of prototype feasibility under the tested conditions rather than as proof of industrial equivalence. Further validation under commercial-scale throughput, continuous feeding, variable potato varieties, wet soil adhesion, dust, and long-term operation is still required.

### 4.2. Effects of Conveyor Speed, Material Spacing, and Threshold

The range analysis and ANOVA jointly showed that conveyor speed had the greatest contribution to overall sorting accuracy, followed by material spacing and classification threshold. Under the additive orthogonal model, all three factors significantly affected overall sorting accuracy (*p* < 0.001). However, for comprehensive mineral-impurity rejection accuracy, the classification threshold showed the largest contribution ratio, indicating that the threshold mainly controlled the balance between impurity missed rejection and potato tuber false rejection. Therefore, the final operating condition should be interpreted as a compromise between overall dynamic sorting stability and mineral-impurity rejection performance. This result is consistent with the operating mechanism of the proposed system, because higher conveyor speed shortens the available time window for image acquisition, recognition, information transmission, and pneumatic actuation, thereby increasing the sensitivity of the system to temporal fluctuation and control error. Material spacing had the second largest effect, because wider spacing reduced target overlap, boundary adhesion, and mutual interference during both image acquisition and rejection. By contrast, the classification threshold had a smaller effect on overall accuracy but played an important role in balancing potato false rejection and impurity missed detection. Therefore, the final operating condition should be interpreted as a trade-off among temporal coordination, target separability, and decision sensitivity rather than the effect of any single factor alone.

Although the optimal conveyor speed identified in this prototype was 0.2 m/s, this value should not be interpreted as the upper limit for industrial application. Instead, it represents the best accuracy–stability trade-off under the current laboratory-scale conveyor, material-spacing condition, PLC timing strategy, and pneumatic rejection mechanism. At the optimal material spacing of 9 cm, the theoretical single-lane material flow rate at 0.2 m/s is approximately 2.22 items s^−1^, corresponding to about 8000 items h^−1^ under ideal continuous feeding. However, the actual throughput in commercial operation would also depend on feeding uniformity, lane number, conveyor width, actuator response speed, and rejection layout. Therefore, for industrial-scale implementation, throughput could be improved by using wider or multi-lane conveyors, faster pneumatic or servo-driven rejection mechanisms, higher-frame-rate image acquisition, and optimized PLC triggering logic. From an engineering perspective, these scalability strategies would affect the accuracy–throughput balance by changing feeding uniformity, image acquisition stability, control timing, and actuator response, and should therefore be evaluated as an integrated system rather than as independent hardware upgrades.

### 4.3. Limitations of the Current System

Several limitations of the present study should be acknowledged. First, the dataset was established under laboratory conditions and contained only three target categories, namely potato tubers, soil clods, and stones; therefore, the diversity of real postharvest environments may not be fully represented. In addition, the potato tuber samples were obtained from locally cultivated yellow-fleshed commercial potatoes in Inner Mongolia, and the diversity of potato varieties was limited. Biological variations among cultivars, including skin color, tuber shape, surface texture, eye depth, and soil adhesion characteristics, may affect detection precision when the system is applied to commercial sorting lines. In particular, uncontrolled industrial or field conditions, such as strong illumination changes, dust interference, wet soil adhesion, severe target overlap, conveyor vibration, and long-term continuous operation, were not fully covered in the present dataset. This limitation is particularly important under wet heavy-clay soil conditions. When potato tubers are heavily covered by wet clay, for example when most of the tuber surface is masked by adhered soil, the visual differences between a soil-covered potato tuber and a soil clod may become very small. Under such extreme conditions, the model may rely mainly on residual contour, size, local shape, and weak texture cues, and its detection precision may decrease. Therefore, the present results should not be overgeneralized to cases in which potato tubers are almost completely covered by wet clay. Future work should include more samples with different clay moisture levels and soil-coverage ratios, and may combine RGB images with depth, multispectral, or weight-related information to improve discrimination between heavily soil-covered tubers and soil clods. Second, the experiments were conducted on a single prototype platform with a fixed camera configuration, control architecture, and pneumatic rejection mechanism, and the quantitative results may therefore depend to some extent on the current system layout. In addition, the long-term endurance of the pneumatic rejection mechanism was not evaluated in this study. In practical postharvest environments, dust accumulation on photoelectric sensors or camera windows may reduce triggering and imaging reliability, while air-pressure fluctuation, pipeline leakage, or valve response variation may reduce the consistency of pneumatic rejection. Although stainless-steel finger flippers were used to improve mechanical strength and resistance to wear, they may still experience surface wear, deformation, or loosening during long-term impact with stones and compacted soil clods. Therefore, routine maintenance would be required in commercial operation, including cleaning sensors and optical windows, checking air pressure and pipeline sealing, inspecting cylinder response consistency, and replacing or adjusting worn finger flippers. Future work should include long-duration endurance tests to quantify actuator reliability, finger-flipper wear, and maintenance intervals under dusty and continuous operating conditions. Third, the temporal–spatial coupling model was developed as an engineering approximation and was mainly intended for relative comparison among candidate models rather than exhaustive characterization of all execution errors. The predicted positional errors were derived from inference-latency fluctuation and conveyor speed, but they were not directly verified by measuring actual rejection-position deviations under multiple conveyor speeds. Future work should therefore include high-speed imaging or position-tracking experiments to compare predicted and measured rejection errors under different operating conditions. Finally, the validation experiments were performed under laboratory conditions, and further field-scale verification under more complex operating scenarios is still needed. Future work should therefore focus on extending the dataset under more diverse field conditions, improving robustness to more complex impurity morphology and illumination variation, and conducting long-term validation in practical postharvest processing environments. In addition, future work may evaluate newer YOLO iterations for more efficient edge deployment. Although YOLOv10n was selected in this study because it provided the most suitable balance among detection accuracy, inference speed, and latency stability under the present hardware and dataset conditions, later YOLO models such as YOLO11 and YOLO26 may provide further opportunities for deployment optimization. YOLO11 has been reported as a more recent Ultralytics YOLO model with improved accuracy, speed, and efficiency, whereas YOLO26 emphasizes end-to-end NMS-free inference, simplified deployment, and faster CPU inference for edge and low-power devices. These features may reduce hardware cost and improve deployment flexibility in future potato tuber–mineral impurity sorting systems. However, their suitability for the present task should be verified experimentally under the same dataset, hardware, and conveyor-based rejection conditions before drawing direct performance conclusions.

## 5. Conclusions

An intelligent sorting system integrating machine vision, a programmable logic controller, and pneumatic rejection was developed to separate potato tubers from mineral impurities, mainly soil clods and stones, under dynamic postharvest conveying conditions. A temporal–spatial coupling model was established to quantify the effect of inference-latency fluctuation on rejection-position deviation. Based on the joint consideration of detection accuracy, inference efficiency, and temporal stability, YOLOv10n was selected as the backbone network, and the final deployment model YOLOv10n-PB was constructed by incorporating a PSA module and dynamic blur augmentation. Under the present test conditions, YOLOv10n-PB achieved a mAP@0.5 of 98.9%, while YOLOv10n showed the most favorable balance between latency stability and deployment efficiency among the candidate models. The orthogonal experiments and ANOVA further showed that conveyor speed had the largest contribution to overall sorting accuracy, whereas classification threshold had the largest contribution to comprehensive mineral-impurity rejection accuracy. This parameter combination represents the accuracy–stability optimum of the current laboratory-scale prototype rather than the maximum achievable throughput for industrial implementation. Validation experiments under these conditions yielded a potato tuber false rejection rate of 1.7%, a comprehensive impurity-rejection accuracy of 98.3%, and an overall sorting accuracy of 98.3%. These results demonstrate that the proposed system can achieve accurate and stable automatic sorting of potato tubers and mineral impurities under representative dynamic operating conditions.

## Figures and Tables

**Table 1:** Latency Measurement Results for Each Component of the System.

Delay Term	Description	Mean (ms)	SD (ms)	Minimum (ms)	Maximum (ms)
$t_{\mathrm{infer}}$	Model inference delay (taking YOLOv10n as an example)	2.2	0.1	2.0	2.5
$t_{\mathrm{comm}}$	Communication delay between host computer and PLC	3.5	0.4	3.0	4.4
$t_{\mathrm{PLC}}$	PLC processing delay	8.0	1.0	7.0	10.0
$t_{\mathrm{cylinder}}$	Pneumatic actuator response delay	28.0	2.5	25.0	33.0
$t_{\mathrm{advance}}$	Total trigger advance time	41.7	2.8	37.5	48.0

**Table 2:** Comparison of Inference Latency Among Mainstream YOLO Models.

Model	Parameters (M)	Mean Latency (ms)	Latency SD (ms)	Inference Speed (f·s^−1^)	Positional Error (mm)	Criterion Met
YOLOv5nu [11]	2.6	8.3	0.6	120	±0.9	No
YOLOv8n [12]	3.2	6.9	0.5	145	±0.75	No
CBAM-YOLOv8n [14]	3.4	9.1	0.7	110	±1.05	No
YOLOv9t [15]	2.0	8.3	0.6	120	±0.9	No
YOLOv10n [16]	2.3	2.2	0.1	450	±0.15	Yes

**Table 3:** Factors and levels used in the orthogonal experimental design.

Level	A: Conveyor Speed (m·s^−1^)	B: Material Spacing (cm)	C: Classification Threshold
1	0.2	1	0.3
2	0.3	3	0.4
3	0.4	5	0.5
4	0.5	7	0.6
5	0.6	9	0.7

**Table 4:** Performance Evaluation of Five Algorithms.

Model	Precision (%)	Recall (%)	mAP@0.5 (%)	Parameters (M)
YOLOv5nu	95.6	97.5	98.4	2.6
YOLOv8n	96.3	97.1	98.1	3.2
CBAM-YOLOv8n	96.7	97.5	98.5	3.4
YOLOv9t	97.8	97.0	99.1	2.0
YOLOv10n	97.1	97.7	98.9	2.3

**Table 5:** Ablation results of different YOLOv10n-based model variants.

Model Variant	Modification	mAP@0.5 (%)	Inference Speed (f·s^−1^)
YOLOv10n-B	Baseline model	97.6	470
YOLOv10n-P	Baseline + PSA	98.3	455
YOLOv10n-PB	Baseline + PSA + dynamic blur aug	98.9	450

**Table 6:** Orthogonal experimental design and corresponding sorting results.

Test No.	Conveyor Speed (m·s^−1^)	Material Spacing (cm)	Classification Threshold	Comprehensive Impurity-Rejection Accuracy (%)	Overall Sorting Accuracy (%)
1	0.2	1	0.3	93.3	92.5
2	0.2	3	0.4	98.3	97.5
3	0.2	5	0.5	96.7	95.8
4	0.2	7	0.6	93.3	95.0
5	0.2	9	0.7	90.0	94.2
6	0.3	1	0.4	93.3	91.7
7	0.3	3	0.5	95.0	94.2
8	0.3	5	0.6	91.7	92.5
9	0.3	7	0.7	86.7	91.7
10	0.3	9	0.3	98.3	97.5
11	0.4	1	0.5	90.0	88.3
12	0.4	3	0.6	88.3	89.2
13	0.4	5	0.7	83.3	88.3
14	0.4	7	0.3	96.7	95.0
15	0.4	9	0.4	98.3	97.5
16	0.5	1	0.6	83.3	85.0
17	0.5	3	0.7	80.0	85.0
18	0.5	5	0.3	93.3	90.8
19	0.5	7	0.4	95.0	94.2
20	0.5	9	0.5	96.7	95.0
21	0.6	1	0.7	73.3	77.5
22	0.6	3	0.3	88.3	85.8
23	0.6	5	0.4	91.7	90.0
24	0.6	7	0.5	90.0	88.3
25	0.6	9	0.6	90.0	91.7

**Table 7:** Range analysis of orthogonal experimental factors based on overall sorting accuracy.

Statistic	A: Conveyor Speed (m·s^−1^)	B: Material Spacing (cm)	C: Classification Threshold
k1	95.00	87.00	92.32
k2	93.52	90.34	94.18
k3	91.66	91.48	92.32
k4	90.00	92.84	90.68
k5	86.66	95.18	87.34
R	8.34	8.18	6.84

**Table 8:** ANOVA results for orthogonal experimental factors based on overall sorting accuracy and comprehensive mineral-impurity rejection accuracy.

(**a**) overall sorting accuracy						
**Factor**	**SS**	**df**	**MS**	**F Value**	***p*** ** Value**	**Contribution (%)**
A: Conveyor speed	209.72	4	52.43	50.38	<0.001	38.94
B: Material spacing	184.23	4	46.06	44.26	<0.001	34.21
C: Classification threshold	132.09	4	33.02	31.73	<0.001	24.53
Error	12.49	12	1.04	—	—	2.32
Total	538.53	24	—	—	—	100.00
(**b**) Comprehensive mineral-impurity rejection accuracy						
**Factor**	**SS**	**df**	**MS**	**F Value**	***p*** ** Value**	**Contribution (%)**
A: Conveyor speed	178.78	4	44.69	51.85	<0.001	19.83
B: Material spacing	176.78	4	44.20	51.27	<0.001	19.61
C: Classification threshold	535.51	4	133.88	155.32	<0.001	59.41
Error	10.34	12	0.86	—	—	1.15
Total	901.42	24	—	—	—	100.00

**Table 9:** Validation results of the optimal parameter combination.

Sample Category	Initial Count	Accepted Count	Rejected Count	Result
Potato	60	59	1	False rejection rate = 1.7%
Soil clod	35	1	34	Rejection accuracy = 97.1%
Stone	25	0	25	Rejection accuracy = 100.0%

## Data Availability

The raw data supporting the conclusions of this article will be made available by the authors on request, due to the full dataset includes large-volume image data, annotation files, and experimental records and is still being organized for long-term archiving. Requests to access the datasets should be directed to the corresponding author at dengwg@imau.edu.cn.
